# Serum Interleukin 17 Levels in Patients with Crohn's Disease: Real Life Data

**DOI:** 10.1155/2014/690853

**Published:** 2014-07-16

**Authors:** Abdurrahman Sahin, Turan Calhan, Mustafa Cengiz, Resul Kahraman, Kubra Aydin, Kamil Ozdil, May Korachi, H. Mehmet Sokmen

**Affiliations:** ^1^Department of Gastroenterology, Elazig Education and Research Hospital, Rizaiye Mah. Inonu Caddesi, 23200 Elazig, Turkey; ^2^Gastroenterology Department, Turkiye Gazetesi Hospital, 34381 Istanbul, Turkey; ^3^Department of Gastroenterology, Ankara Oncology Education and Research Hospital, 06500 Ankara, Turkey; ^4^Department of Gastroenterology, Batman State Hospital, 72070 Batman, Turkey; ^5^Department of Genetics and Bioengineering, Yeditepe University, 34755 Istanbul, Turkey; ^6^Department of Gastroenterology, Umraniye Training and Research Hospital, 34764 Istanbul, Turkey

## Abstract

The aim of this study was to investigate serum IL17 levels in patients with Crohn's disease (CD) and to investigate the relationship between serum IL17 levels with disease activity. *Methods*. Fifty patients with CD and sex- and age-matched 40 healthy controls were included in the study. The serum IL17 levels, complete blood count, blood chemistry, erythrocyte sedimentation rate (ESR), and C-reactive protein (CRP) levels were measured, and Crohn's disease activity was calculated using Crohn's disease activity index (CDAI). *Results*. The mean serum IL17 level of CD patients did not differ from those of healthy controls (*P* > 0.05). There was no difference between the mean serum IL levels of active CD patients and of quiescent CD patients (*P* > 0.05). However, the mean IL17 level of active patients was lower than of control subjects (*P* = 0.02). Serum IL17 was not correlated with inflammatory markers (ESR, CRP, white blood count, platelet count, and albumin) and CDAI. *Conclusions*. Peripheral blood serum IL17 levels of CD patients were not higher than of healthy controls, and also, serum IL17 level was not correlated with clinical disease activity. Peripheral IL17 measurement is not a useful tool for detecting and monitoring Crohn's disease which is understood to have complex etiopathogenesis.

## 1. Introduction

Crohn's disease (CD) is a chronic relapsing inflammatory disease affecting the gastrointestinal tract and presenting with extraintestinal manifestations as well. Although the etiopathogenesis of this disease is not completely understood, it seems to be influenced by several environmental factors in genetically predisposed individuals [1, 2]. T helper lymphocytes (Th) play an important role in the pathogenesis of CD. Crohn's disease is postulated to be an inflammatory disease mediated by Th1 cells and recently also by Th17 cells [3].

Th17 cells are major contributors to several autoimmune diseases that were previously thought to be Th1 cell predominant diseases. These autoimmune diseases can be listed as rheumatoid arthritis, psoriasis, systemic lupus, scleroderma, multiple sclerosis, inflammatory bowel disease, autoimmune myocarditis, and endometriosis [4]. Th17 cells also play an important role in maintaining intestinal mucosal barrier function by affecting innate and adaptive responses. Mucosal Th17 cells prevent migration of pathogens from breaking mucosa to the systemic circulation through the chemotaxis of neutrophils and macrophages.

One of the important features of Th17 cells is to balance mucosal inflammation by regulating the immunogenic response against self-antigens or intestinal pathogens due to their relationship with regulatory T cells. The other is plasticity, the ability of these cells to differentiate to other T cell subgroups under various types of stimulation. Th17 cells that are induced in vitro by transforming growth factor *β* (TGF*β*) and interleukin (IL) 6 alone produce both IL17 and the inhibitory cytokine IL10 and do not induce inflammation, whereas Th17 cells that have been stimulated with IL23 after stimulation with TGF*β* and IL-6 produce other proinflammatory cytokines and chemokines, but not IL-10, and are involved in inflammation [5]. The cytokines secreted by Th17 cells are IL21, IL23, IL26, tumor necrosis factor *α*, interferon *γ*, and mainly IL17 [6].

IL17 is produced by T cells, macrophages, dendritic cells, natural killer, and other T cells. Thereby, IL17 influences several types of cells of the immune system, particularly B cells and antigen presenting cells, to induce the expression of several cytokines, chemokines, chemokine receptors, and metalloproteases [4, 7, 8]. The role of Th17 cells and IL17 cytokines in CD is not completely understood, and data on this issue has been obtained from animal models and tissue sample studies from inflamed mucosa [9–11]. On the other hand, the IL17 level in sera of CD patients has not yet been studied. In the present study, IL17 levels of CD patients were investigated. The relation of IL17 levels with disease activity and other proinflammatory markers was also evaluated.

## 2. Materials and Methods

### 2.1. Patients

Fifty patients diagnosed with CD and 40 controls were included in the study between January 2012 and August 2012. Demographic characteristics, disease features (location, behavior, disease duration, and extraintestinal manifestations), medications, and disease activity of CD patients were recorded. Disease activity in CD patients was assessed by the Crohn disease activity index (CDAI) [12]. A score under 150 was accepted as inactive disease, while a CDAI score over 150 was considered as active disease [13]. The medications were classified as “tumor necrosis factor blocker (anti-TNF)” group (anti-TNF with/without immunomodulatory and with/without mesalazine), immunomodulatory group (immunomodulatory drug with/without corticosteroids and with/without mesalazine), and mesalazine group (mesalazine with/without corticosteroids). The control group consisted of 40 age- and sex-matched subjects coming to the hospital with dyspeptic complaints. Subjects who had any inflammatory disease or disorder with a characteristic of changed IL17 levels were excluded from the control group. Pregnancy and age younger than 18 years old were other exclusion criteria for patients and controls. The protocol was approved by the local ethics committee. Informed consent was obtained from all subjects before obtaining samples. Samples of peripheral blood were allowed to clot and were then centrifuged. The sera were frozen at −80°C immediately after sample collection.

### 2.2. Measurement of Enzyme-Linked Immunosorbent Assay (ELISA) for Human IL17

IL17 levels of samples were assayed with a human IL17 ELISA kit (EastBiopharm, Hangzhou, China) according to the manufacturer's instructions. 50 *μ*L of standard and serum samples were added to the wells. 50 *μ*L of biotinylated antibodies was then added to each well and incubated. Two hours later, wells were washed and a 100 *μ*L solution of streptavidin-HRP was added and incubated at room temperature for one hour. Standard curves were then calculated at 450 nm with a spectrophotometer by an ELISA technique. The limit of detection was 2 ng/L.

### 2.3. Statistical Analyses

Statistical Package for Social Sciences (SPSS) version 11.0 was used for analysis. Normally distributed descriptive variables were expressed as mean ± SD; variables with skewed distribution were expressed as median and range (minimum and maximum). Student's *t*-test was used for comparison of normally distributed continuous variables; Wilcoxon rank-sum test was used if the distribution was not normal. In the comparison of more than two groups, ANOVA was used for parametric variables and Kruskal-Wallis test was used for nonparametric variables. Correlation was tested by Pearson or Spearman correlation tests where appropriate. Statistical analyses were performed using the Mann-Whitney *U* test and Spearman rank correlation test. Differences resulting in *P* values less than 0.05 were considered statistically significant.

## 3. Results

**Figure 1:**
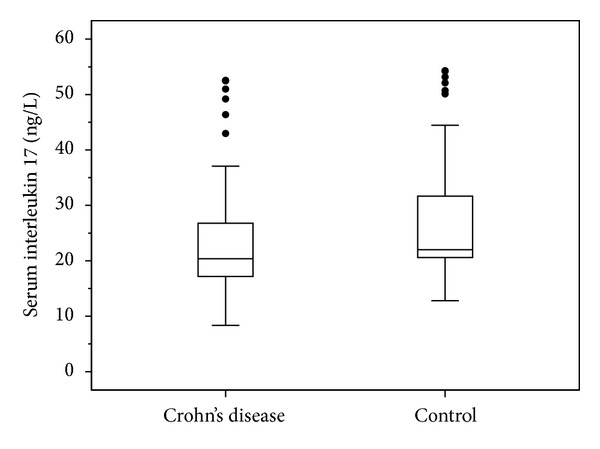
Box plot of serum IL17 levels of Crohn's patients and controls.

A total of 50 CD patients (25 male/25 female) and 40 control subjects (19 male/21 female) were recruited into the study. There was no difference in age (38.5 for CD patients versus 39.0 for controls; *P* > 0.05) and gender. Mean erythrocyte sedimentation rate (ESR), C-reactive protein (CRP), and platelet counts of CD patients were higher than in control subjects. On the other hand, albumin was lower in the CD group. There was no significant difference in IL17 levels between subjects with CD and healthy subjects (24.29 ± 11.03 versus 27.93 ± 12.07; *P* > 0.05) (Figure 1). Demographic and laboratory data for CD patients and control subjects are shown in Table 1 and serum IL17 levels of patients and controls are presented in Figure 1.

Twenty-one patients with CD had ileal disease while 27 patients had ileocolonic disease. Only two patients had colonic disease. Eighteen of the CD patients had penetrating disease behavior. On the other hand, only three patients had stricturing disease. There was no difference in IL17 levels between groups according to disease location and disease behavior (Table 2). Twenty patients with CD had active disease in the assessment period. Median CDAI was 124.5 (1–394). The IL17 levels of active patients and patients in remission were similar (23.82 ± 11.12 ng/L versus 24.61 ± 11.06 ng/L, resp.; *P* > 0.05). The mean IL17 level of active patients was lower than of control subjects (23.82 ± 11.12 ng/L versus 27.93 ± 12.07 ng/L; *P* = 0.02), while no difference was found between IL17 levels of CD patients in remission and control subjects. Serum IL17 levels of treatment groups were similar. Moreover, serum IL17 levels of patients taking immunosuppressive treatment (immunomodulatory group and anti-TNF group) and of patients taking only mesalazine did not differ (Table 2).

There was no relationship between IL17 and CDAI. A correlation between IL17 and other proinflammatory markers, such as ESR, CRP, WBC count, platelet count, hemoglobin, and albumin, was not found. CDAI was correlated with CRP (*P*: 0.03, *r* ^2^: 0.300). Hemoglobin and albumin were also negatively correlated with CDAI (*P*: <0.01, *r* ^2^: −0.411 and P: <0.01, *r* ^2^: −0.601, resp.).

## 4. Discussion

After the discovery of Th17 cells and the importance of these cells in the pathogenesis of CD, secreted cytokines from these cells and pathways that are involved in Th17 cells have been considered as novel targets for disease activity monitoring and have therapeutic implications. In previous studies it was demonstrated that Th17 and IL17 levels were increased in inflamed mucosa. Veny et al. found that blood IL17 production in active CD patients was higher than in CD patients in remission and in healthy subjects [14]. On the other hand, serum mononuclear cell expression of IL17 was higher for both active and inactive Crohn's disease patients compared to healthy subjects, and IL17 expression of blood mononuclear cells was correlated with disease activity. In the same study, it was shown that patients with active disease with longer duration (more than 1 year since diagnosis) showed a significant increase in the percentage of IL17 producing T cells and blood IL17 production compared with early active patients (within 32 weeks of disease onset), inactive patients, and healthy subjects. In another previous study, IL17 expression was demonstrated in colonic mucosa of CD and ulcerative colitis (UC), while it was not found in colonic mucosa samples of infectious colitis, ischemic colitis, and healthy subjects [9]. They also found elevated serum IL17 levels only in 10 active CD patients and 10 active UC patients. Patients who were recruited into this study only received salicylate treatment. In contrast to the findings of the abovementioned studies, we found in the present study that circulating IL17 levels of CD patients were not higher than control subjects. Moreover, active CD patients had lower serum IL17 levels.

Th17 cells and IL17 contribute to inflammatory pathways in many rheumatologic and autoimmune diseases, such as rheumatoid arthritis, systemic sclerosis, systemic lupus erythematosus, Sjögren syndrome, psoriasis, familial mediterranean fever, multiple sclerosis, and ulcerative colitis [4]. Interleukin 17 levels were found to be higher in sera of patients with these autoimmune diseases [4, 15, 16]. Moreover, it was shown that immunomodulatory treatments suppress IL17. In a study of patients with rheumatoid arthritis, it was shown that serum IL17 levels were decreased after treatment with adalimumab and methotrexate [17]. Another study in patients with rheumatoid arthritis showed that baseline serum IL17 levels of patients were higher than of healthy subjects [18]. After anti-TNF treatment, peripheral blood Th17 cell count, serum IL17 level, and serum TNF level were decreased in the treatment responsive group. On the other hand, while serum TNF level was decreased, peripheral Th17 cell count and serum IL17 level were increased in nonresponders. These studies showed that immunosuppressive therapies influence serum IL17 level in treatment responsive patients, and this explains why serum IL17 levels of CD patients did not differ from serum IL17 levels of healthy subjects.

Crohn's disease emerges as a complex, multigenic disease that is identified with both frequent mutations and rare mutations, rather than high penetrance mutations in gene analysis [19]. This genetic heterogeneity leads to different phenotypic presentations of CD acting on many different cells and distinct pathways, not all of them present in all patients [20]. A recent study showed that anti-TNF therapy suppresses intestinal inflammation in IBD patients via downregulation of macrophage and Th17 pathway genes [21]. In this study, a significant portion of the patients had taken immunosuppressive agents (68%) when they were recruited into the study. Only six patients' IL17 levels were over 40 ng/L; two of them were on anti-TNF treatment and one of them was a newly diagnosed patient (Figure 1). From ten CD patients taking an anti-TNF drug, only two patients had higher IL17 levels. Our results suggest that anti-TNF treatment fails to suppress Th17 driven inflammation in some CD patients (nonresponders).

An interesting result of this study was that active CD patients had lower serum IL17 levels than control subjects. This might be related to the fact that active CD patients had mild or moderately active disease. Another reason is that IL17 producing inflammatory cells might be effectively controlled by immunosuppressive agents. We suggest that serum IL 17 level is not a good indicator for differentiating active disease from inactive disease, and perhaps it is not an accurate marker for monitoring disease activity. Further studies are needed with serial IL17 measurements for evaluating serum IL17 as a disease monitoring marker. In addition, further studies are needed with larger series to evaluate serum IL17 levels in severely active patients and to compare them with healthy subjects.

Earlier studies demonstrated Th17 stimulation, IL17 related mRNA expression, and IL17 production in inflamed mucosa samples of CD and UC patients [10, 22–24]. There is limited data concerning serum IL17 levels in IBD patients. In a recent study, serum IL17 levels of UC patients were found to be higher than in healthy subjects [25]. In contrast to the result of the study with UC patients, we did not find any difference in IL 17 levels between CD patients and healthy subjects. Furthermore, we also found that there was no difference between active CD patients and inactive CD patients. Schwarzmaier et al. showed in a study evaluating peripheral monocyte functions that IL17 expression and release, in comparison to other proinflammatory cytokine expression and release, did not differ between inactive CD patients and healthy controls [26]. Inactive patients recruited into this study were receiving only salicylate or tapering budesonide without any immunomodulatory drug or anti-TNF drug. An increase in the Th17 population in inflamed mucosa and peripheral blood mononuclear cell culture was demonstrated in earlier studies [27]. Surprisingly, our results did not establish a similar increase in serum IL17 levels. This finding may be because serum IL17 might not be a systemically effective mediator. Instead, IL17 acts as a trigger of proinflammatory responses at the inflammation site in the intestine [10, 28].

Serum IL17 levels were not related to CDAI and other inflammatory serum markers such as ESR, CRP, albumin, and hemoglobin. On the other hand, CDAI was correlated with hemoglobin, CRP, and albumin. This finding shows that serum IL17 levels are not suitable for determining disease activity. Instead, hemoglobin, CRP, and albumin are more suitable tests. Crohn's disease activity index is a compound index containing clinical laboratory parameters and predominantly clinical features. However, CDAI score does not reflect ongoing intestinal inflammation sufficiently. This explains why CDAI is not correlated with inflammatory markers and proinflammatory cytokines, like interleukin 17.

Consequently, although the importance of Th17 cells has been well defined in Crohn's disease etiopathogenesis, IL17, the main cytokine derived from these cells, does not increase in the sera of CD patients. IL17 measurement is not a suitable test to differentiate CD patients from healthy controls and also active CD patients from inactive CD patients.

## Figures and Tables

**Table 1:** Baseline characteristics and laboratory values of patients with Crohn's disease and control subjects.

	Crohn's disease	Control group	*P*
Age median (min.–max.) years	38.5 (18–63)	39 (22–60)	0.84
Gender (female/male)	25/25	21/19	0.81
Montreal classification *n* (%)			
L1 (isolated ileal disease)	21	—	
L2 (isolated colonic disease)	2	—	
L3 (ileocolonic disease)	27	—	
L4 (concomitant UGI disease)	0	—	
B1 (nonstricturing and nonpenetrating)	29	—	
B2 (stricturing)	3	—	
B3 (penetrating)	18	—	
CDAI median (min.–max.)	124.5 (1–394)	—	
Treatment *n* (%)			
Mesalazine	46 (92%)	—	
Corticosteroid	11 (22%)	—	
Azathioprine	27 (54%)	—	
Methotrexate	1 (2%)	—	
Anti-TNF	12 (24%)	—	
White blood cells (/mm^3^)	7410 ± 1760	7170 ± 2540	0.60
Hemoglobin (g/dL)	13.2 ± 2.0	13.8 ± 1.7	0.13
Platelets (/mm^3^)	314000 ± 86500	274000 ± 60500	**0.01**
Glucose (mg/dL)	94 ± 16	92 ± 8	0.35
Blood urea nitrogen (mg/dL)	24.4 ± 8.5	25.8 ± 7.9	0.42
Creatinine (mg/dL)	0.73 ± 0.20	0.75 ± 0.13	0.56
ALT (U/L)	19.7 ± 15.3	25.4 ± 19.8	0.13
Albumin (g/dL)	4.1 ± 0.7	4.6 ± 0.3	**<0.01**
ESR (mm/hour)	25.8 ± 22.8	15.8 ± 11.3	**0.01**
CRP (mg/dL)	1.56 ± 3.38	0.50 ± 0.39	**0.03**
IL17 (ng/L)	24.29 ± 11.03	27.93 ± 12.07	0.14

**Table 2:** Disease features and medications of Crohn's disease patients and assessment according to IL17 measurements.

	*n* (%)	IL17 (ng/L)	*P*
Activity			>0.05
Active	20	23.82 ± 11.12	
Inactive	30	24.61 ± 11.06	
Behavior *n* (%)			>0.05
Nonstricturing and nonpenetrating	29	24.07 ± 11.11	
Penetrating	18	24.40 ± 11.94	
Stricturing	3	25.83 ± 6.26	
Location			>0.05
Ileal	21	22.66 ± 8.30	
Ileocolonic	27	25.87 ± 12.07	
Colonic	2	30.50 ± 22.45	
Treatment groups			>0.05
Mesalazine group	16	24.56 ± 11.72	
Immunomodulatory group	22	23.62 ± 10.39	
Anti-TNF group	12	25.18 ± 12.13	
Immunomodulatory + anti-TNF	34	24.31 ± 11.01	
